# CTCF-associated chromatin changes between *HOXD1* and *HOXD8* contribute to TNBC progression

**DOI:** 10.1038/s12276-026-01861-6

**Published:** 2026-10-06

**Authors:** Ji Hoon Oh, Da Som Jeong, Clara Yuri Kim

**Affiliations:** 1 Department of Biological Sciences, Keimyung University College of Natural Sciences, Daegu, Republic of Korea; 2 Department of Internal Medicine, Yonsei University College of Medicine, Seoul, Republic of Korea; 3 Yonsei Institute for Digital Health, Yonsei University, Seoul, Republic of Korea; 4 Department of Genetics, Perelman School of Medicine, University of Pennsylvania, Philadelphia, PA USA

**Keywords:** Breast cancer, Prognostic markers

## Abstract

Homeobox (HOX) genes and their encoded DNA-binding homeoproteins are important regulators of developmental processes and have been implicated in cancer pathogenesis. Here, we investigated the roles of *HOXD1* and *HOXD8* across different molecular subtypes of human breast cancer (BC) using a combination of in silico analyses of large-scale patient datasets, in vitro RNA interference (RNAi) phenotyping, and in vivo validation studies. Expression levels of *HOXD1* and *HOXD8* were differentially altered across BC subtypes and were specifically reduced in the aggressive triple-negative breast cancer (TNBC) subtype. Functional analyses demonstrated that reduced *HOXD1* ﻿and *HOXD8* expression was linked to enhanced invasive, migratory, and proliferative phenotypes. In addition, our molecular analyses suggest that CCCTC-binding factor (CTCF)-associated chromatin changes and DNA methylation status at the HOXD locus contribute to coordinated regulation of *HOXD1* and *HOXD8* expression in BC cells. Collectively, our findings support a potential tumor-suppressive role for *HOXD1* and *HOXD8* in TNBC and suggest that the *HOXD1*/*HOXD8* regulatory axis has biological and therapeutic relevance in aggressive BC.

## HOXD1 and HOXD8 roles in triple-negative breast cancer

Triple-negative breast cancer (TNBC) is a challenging subtype of breast cancer because of its aggressive nature and lack of targeted therapies. This study identifies a critical regulatory axis involving HOXD1, HOXD8, and CTCF, which is suppressed in TNBC. Researchers used various breast cancer cell lines and mouse models to explore the chromatin-associated regulatory mechanisms of *HOXD1* and *HOXD8*. They found that these genes are downregulated in TNBC, contributing to aggressive cancer phenotypes by affecting *FOXA1* expression. The study demonstrated that restoring *HOXD1* and *HOXD8* expression reduced tumor aggressiveness, whereas deleting CTCF-binding sites increased tumor progression. These findings highlight the importance of chromatin architecture in gene regulation and suggest that targeting this regulatory axis could offer new therapeutic strategies for TNBC. Future research could further explore the role of chromatin interactions and epigenetic modifications in TNBC progression.

This summary was initially drafted using artificial intelligence, then revised and fact-checked by the author.

## Introduction

Breast cancer (BC) remains one of the most frequently diagnosed cancers and a leading cause of female cancer mortality worldwide^1,2^. BC typically develops from the ducts or lobules of the breast tissue and is classified into four major molecular subtypes based on the expression of hormone receptors: luminal A (ER/PR+, HER2−), luminal B (ER/PR+, HER2+), HER2-enriched (ER/PR−, HER2+), and triple-negative (ER/PR−, HER2−) breast cancers (TNBC)^3^. TNBC accounts for approximately 10–15% of all BCs and exhibits the most aggressive clinical features, characterized by rapid growth and high metastatic potential^4,5^. A major challenge in treating TNBC is the absence of ER, PR, and HER2 proteins, which renders endocrine therapy and targeted drugs ineffective^6,7^. Therefore, current treatment relies primarily on surgery and chemotherapy, but the high recurrence rate within a short-term (3–5 years) highlights the limitations of existing therapies^8,9^. Consequently, new accessible approaches are urgently warranted to identify effective targeted therapies or biomarkers for TNBC.

The homeobox (HOX) gene family, a highly conserved component of the homeobox superfamily, is crucial for body patterning along the anterior–posterior axis during embryonic development^10^. In vertebrates, including humans and mice, there are 39 HOX genes organized into four different clusters: HOXA, HOXB, HOXC, and HOXD^10,11^. The spatiotemporal expression of HOX genes is tightly regulated by a phenomenon known as collinearity, where genes located at the 3′ end of the cluster are expressed earlier and more anteriorly than those at the 5′ end^12,13^. More recently, these highly regulated HOX genes in embryogenesis have been implicated in various cancers, including lung, ovarian, prostate, colon, and breast cancer^14^. Depending on the context, HOX genes can function as oncogenic factors or tumor suppressors, regulating processes such as cancer cell survival, proliferation, motility, and immune response^15–17^. Specifically, the HOXD cluster has been linked to the development and progression of BC, with abnormal expression of *HOXD3*, *HOXD9*, and *HOXD10* previously associated with TNBC tumorigenesis and progression^14^. However, previous studies have primarily focused on HOXD family members associated with invasive or metastatic phenotypes, whereas subtype-specific regulatory patterns and chromatin-associated regulatory mechanisms within the HOXD cluster remain unclear. Therefore, we sought to identify HOXD family members exhibiting TNBC-specific dysregulation and to investigate their chromatin-associated regulatory mechanisms.

The three-dimensional (3D) organization of chromatin is a critical determinant of gene expression, as changes in chromatin architecture can affect the accessibility of DNA to transcription factors and other regulatory proteins, which can have profound effects on cellular processes^18^. For example, during development, cells undergo differentiation in which they become specialized for specific functions. This process involves changes in gene expression patterns that are regulated by architectural changes^19^. Similarly, cancer cells are often characterized by abnormal chromatin architecture, resulting in oncogene dysregulation and tumor suppressor gene silencing, thereby contributing to cancer initiation and progression^20^. Moreover, the aberrant chromatin structure in cancer cells can contribute to genomic instability and promote the acquisition of other hallmarks of cancer, such as increased proliferation, evasion of apoptosis, and resistance to therapy^20,21^.

The CCCTC-binding factor (CTCF) is a highly conserved protein that plays a crucial role in regulating chromatin architecture, genomic organization, and gene expression^22^. CTCF is a versatile protein that can act as an activator, repressor, or insulator depending on the context of its binding site and interacting partners^23^. The dysregulation of CTCF expression or function has been associated with several oncogenic processes, including aberrant chromatin remodeling, altered gene expression, genomic instability, and impaired DNA repair^24^. These findings suggest that CTCF serves a critical role in cancer development and progression, and that targeting its regulation offers a promising therapeutic strategy.

In this study, we demonstrate that coordinated expression of HOXD1 and HOXD8 is associated with altered CTCF occupancy, DNA methylation, and CTCF-associated chromatin changes at the HOXD locus. Importantly, we identified that HOXD1 and HOXD8 are downregulated in TNBC, where they exert tumor-suppressive functions by regulating *FOXA1* expression, thereby controlling invasiveness and metastasis. Furthermore, using a mouse xenograft model, we demonstrated that forced expression of HOXD1 and HOXD8 attenuated TNBC-associated aggressive phenotypes, whereas deletion of specific CTCF-binding sites (CBSs) at the *HOXD1* and *HOXD8* loci enhanced tumor progression in vivo. Together, our findings support a model in which these epigenetic alterations are associated with coordinated downregulation of HOXD1 and HOXD8, potentially contributing to TNBC-associated phenotypes by regulating FOXA1 and providing therapeutic insight into TNBC.

## Materials and methods

### Cell lines

Parental MCF7, T47D, BT474, MDA-MB-453, and MDA-MB-231 cell lines were provided by Drs Yong Nyun Kim and Kyung Tae Kim (National Cancer Center). The MDA-MB-468 cell line was provided by Prof. Jong Hoon Park (Sookmyung Women’s University). These BC cell lines were maintained in Dulbecco’s Modified Eagle Medium (DMEM: LM001-05, WelGENE Inc.) supplemented with 10% fetal bovine serum, 100 mg/ml penicillin, 10 mg/ml streptomycin sulfate, and 25 µg/ml amphotericin B (LS204-01, WelGENE Inc.) at 37 °C in 5% CO_2_. For overexpression studies, full-length *HOXD1* and *HOXD8* cDNAs were cloned into a pCMV6 expression vector (PS100026, OriGene). MDA-MB-231 and MDA-MB-468 cells were transfected with a plasmid DNA using Attractene eagent (301005, Qiagen). For loss-of-function studies, MCF7 and BT474 cells were transfected for 48 hours with 40 nM siHOXD1, 20 nM siHOXD8, or 25 nM siCTCF using G-fectin (all purchased from Genolution Inc.). For CTCF knockdown, ON-TARGET plus CTCF siRNA (L-02016-00-0005) was purchased from Dharmacon, which uses a patented dual-strand modification to reduce off-target effects. Negative control was provided by Genolution Inc. For DNA demethylation experiments, cells were treated with 5-Aza-2′-deoxycytidine (5-AzadC; A3656, Sigma-Aldrich). Luciferase activity was measured using the Dual-Luciferase Reporter Assay System (E1910, Promega), according to the manufacturer’s instructions.

### Real-time quantitative PCR

Real-time quantitative PCR (RT-qPCR) analysis was performed using the StepOnePlu Real-Time PCR System (43-766-00, Applied Biosystems) and Power SYBR Green PCR Master Mix (Applied Biosystems) kits. All samples were analyzed in triplicate, and gene expression levels were normalized to β-actin, which was used as an internal control.

### Western blotting and antibodies

All BC cell lines were harvested and lysed with Nonidet-P40 buffer (150 mM NaCl, 1% NP-40, and 50 nM Tris, pH 8.0). The concentration of lysate was measured using a Pierce BCA Protein Assay Kit (23225, Thermo Scientific). Each sample was loaded onto 8–10% SDS–PAGE, and then electrotransferred to PVDF transfer membranes (1620177, Bio-Rad). Immunoreactive bands were detected using primary antibodies against HOXD1 (ab235103, Abcam), HOXD8 (TA810942, Thermo Scientific), CTCF (#3418, Cell Signaling Technology), and β-actin (ab6276, Abcam), followed by HRP-conjugated anti-rabbit (ab97051, Abcam) or anti-mouse (ab6789, Abcam) secondary antibodies. Immunoblot was visualized using SuperSignal West Pico Chemiluminescent Substrate Kit (34580, Thermo Scientific). β-actin was used as an internal loading control to normalize protein expression levels.

### Transwell invasion assay

For the migration assay, 5 × 10^4^ cells were harvested and resuspended in 500 µl of serum-free DMEM. Cell suspension was seeded onto a cell culture insert (353097, Falcon Corning). For the invasion assay, the cell suspension was seeded on the top of precoated inserts with 150 mg/mL Matrigel (354234, BD, Franklin) mixed with a coating buffer (0.01 M Tris, pH 8.0, and 0.7% NaCl). DMEM with 10% fetal bovine serum was added to the lower chambers of the 24-well plates. After 24–72 hours, the cells in the bottom surface of the inserts were fixed with methanol for 25 minutes on ice, washed with 1x PBS, stained with DAPI, and visualized by fluorescent microscopy. The number of migrated or invaded cells was quantified using ImageJ software (National Institutes of Health).

### Cell proliferation assay

MCF7 and MDA-MB-231 cells were seeded in 96-well plates at a density of 7500 cells/well, with four replicate wells for each experimental group. MCF7 cells transfected with siCON, siHOXD1, or siHOXD8 and MDA-MB-231 cells transfected with control vector, HOXD1 overexpression vector, or HOXD8 overexpression vector were used for cell proliferation analyses. Cell proliferation was assessed using the MTT assay on days 1, 2, 3, and 5 after seeding. MTT reagent (5 mg/ml in PBS; M5655, Sigma-Aldrich) was diluted 1:10 in a culture medium immediately before use and added to each well. After 3 hours of incubation at 37 °C, the medium was removed and the formazan crystals were dissolved in DMSO. Absorbance at 570 nm was measured using a microplate reader.

### Chromatin immunoprecipitation assay

All BC cell lines were harvested at 80–90% confluency with 1 × 10^6^ cells per antibody. The cells were cross-linked with 1% formaldehyde for 15 minutes at room temperature. To quench the reaction, 2.5 M glycine was added to make a final concentration of 125 mM, and the mixture was incubated for 10 minutes at room temperature. Then, cells were washed with cold 1x PBS and lysed for 10 minutes on ice in lysis buffer consisting of 1% SDS, 1% Triton X-100, 0.1% sodium deoxycholate, 10 mM EDTA, 50 mM Tris-HCl (pH 8.0), and a protease inhibitor (11697498001, Merck). Lysates were sonicated to obtain 500–1000 bp DNA fragments, which was confirmed by loading on a 1% agarose gel. Then, the lysates were incubated with Protein A/G PLUS-Agarose beads (sc-2003, Santa Cruz) on a rotator for 1 hour at 4 °C. For DNA–protein complexes, precleared DNA was incubated with corresponding antibodies overnight at 4 °C. Chromatin immunoprecipitation (ChIP)-grade anti-CTCF antibody (2899S, Cell Signaling Technology) and non-immune mouse IgG (sc2025, Santa Cruz) were used for immunoprecipitation. ChIP enrichment levels were quantified by qPCR using locus-specific primer pairs and normalized relative to IgG controls.

### Chromosome conformation capture assay

For the chromosome conformation capture (3C) assay, 1 × 10^7^ cells were harvested and cross-linked with 1% formaldehyde for 15 minutes at room temperature. To quench the reaction, 2.5 M glycine was added, and samples were incubated on ice for 10 minutes. Then, cells were lysed in lysis buffer (10 mM Tris-HCl, pH 7.5, 10 mM NaCl, 0.2% NP-40, 1x protease inhibitor (P8340, Merck)) for 10 minutes on ice. The pelleted nuclei were mixed with 1.2x restriction enzyme buffer, SDS was added to make a final concentration of 0.3%, and then incubated for 1 hour at 37 °C in a rotating incubator. Triton X-100 was added to the lysate to make a final concentration of 2%, and the mixture was incubated for 1 hour at 37 °C in a rotating incubator. Chromatin interaction frequencies were quantified by qPCR using locus-specific primer pairs and normalized against the bacterial artificial chromosome (BAC) control template.

### CRISPR–Cas9-mediated deletion and clone validation

To generate CBS deletion clones, sgRNAs targeting the HOXD1 #2 and HOXD8 #2 CBSs were designed using a CRISPR design tool. Cells were transfected with CRISPR–Cas9 plasmids containing the corresponding sgRNAs, and single-cell clones were isolated by limiting dilution. Genomic DNA from individual clones was extracted, and deletion of the targeted CBS regions was validated by PCR amplification. Potential off-target effects were minimized by selecting sgRNAs with low predicted off-target scores based on in silico analysis.

### Combined bisulfite restriction analysis (COBRA) and bisulfite sequencing

The promoter methylation status of *HOXD1* and *HOXD8* genes was determined by combined bisulfite restriction analysis (COBRA) and bisulfite sequencing. Using the MethPrimer program, primers were designed to amplify a portion of CpG islands and the upstream regions of *HOXD1* and *HOXD8* from bisulfite-modified DNA. Furthermore, 50 ng of DNA was amplified under the following conditions: 5 minutes at 94 °C, 40 cycles of 30 seconds at 94 °C, 30 seconds at 55–58 °C, 1 minute at 72 °C, followed by one cycle of 10 minutes at 72 °C. For COBRA analysis, PCR products were incubated with BstUI restriction enzyme (CGCG) for 4 hours at 60 °C and loaded onto a 1.8% agarose gel. For bisulfite sequencing, PCR products were cloned into the pGEM-T easy vector (Promega) and individual clones were sequenced (COSMO Genetech Co., Ltd).

### Animal experiments

All animal experiments were approved by the Institutional Animal Care and Use Committee of Yonsei University (IACUC number: 2020-0197) and were conducted in compliance with the Guide for the Care and Use of Laboratory Animals. BALB/c nude mice were purchased from Orient Bio Inc. For in vivo xenograft experiments, five mice per experimental group were used. All mice were maintained under 12-hour light and 12-hour dark conditions in a specific pathogen-free barrier facility. For xenograft experiments, 2.0 × 10^6^ MCF7 empty vector control cells (MCF7: Empty vec.), MCF7 cells with deletion of the CBSs at the HOXD1 and HOXD8 loci (MCF7: ΔCBS D1 & D8), ΔCBS cells transfected with empty vector (MCF7: ΔCBS D1 & D8: Empty vec.), and HOXD1/HOXD8-overexpressing ΔCBS cells (MCF7: ΔCBS D1 & D8: HOXD1 & D8 ov.) were suspended in 100 μl PBS/Matrigel mixture (1:1, v/v; BD Biosciences) and injected either into the mammary fat pad or intravenously through the tail vein. Tumor volume was monitored every 2 days using a caliper for 40 days. When the tumor size reached 10 mm^3^, the volume of the tumor (V) was calculated using the following equation: V (mm^3^) = 3.14 × width (W) × length (L) × height (H)/6 of the tumor. After 8 weeks of injection, mice were euthanized and visceral organs were collected for further analysis. In addition, tumor metastasis was evaluated at different time points and compared between mice injected with MCF7: Empty vec., MCF7: ΔCBS D1 & D8, MCF7: ΔCBS D1 & D8: Empty vec., and MCF7: ΔCBS D1 & D8: HOXD1 & D8 ov. cells. Metastasis was monitored using an in vivo imaging system (IVIS; Caliper Life Science) for 5 days. The mice were imaged at different time points of injection (12, 24, 48, 72, 96, and 120 hours), euthanized, and dissected to collect visceral organs for metastasis analysis.

### Bioinformatics analysis

Survival analysis was performed using the Kaplan–Meier plotter (http://kmplot.com). The web-accessible database cBioPortal (https://www.cbioportal.org) was used to determine the expression patterns of HOXD cluster genes in various BC subtypes (The Cancer Genome Atlas (TCGA), Nature 2012). The web-accessible database Cancer Cell Line Encyclopedia (CCLE; https://sites.broadinstitute.org/ccle) was used to determine any positive correlation between *HOXD1* and *HOXD8* in BC cells. The GEO dataset (GSE65194), consisting of 11 normal breast tissues and 144 BC tissue samples^25^, was used to evaluate the expression patterns of *HOXD1* and *HOXD8* in patients with BC. Public chromatin interaction and occupancy datasets, including ChIA–PET, ChIP-seq (GSE146822), and chromatin interaction tracks, were obtained from publicly available UCSC Genome Browser and ENCODE resources.

### Statistical analysis

All quantitative experiments were independently performed at least three times unless otherwise indicated. Data are presented as mean ± s.e.m. Statistical significance was determined using Student’s *t*-test, one-way anlaysis of variance (ANOVA), or chi-squared test, as appropriate. A *P*-value of <0.05 was considered statistically significant. Comparisons were performed relative to the corresponding control groups, including siCON, Empty vec., or WT controls, as indicated in each experiment.

## Results

### Downregulation of *HOXD1* and *HOXD8* correlates with TNBC

**Fig. 1:**
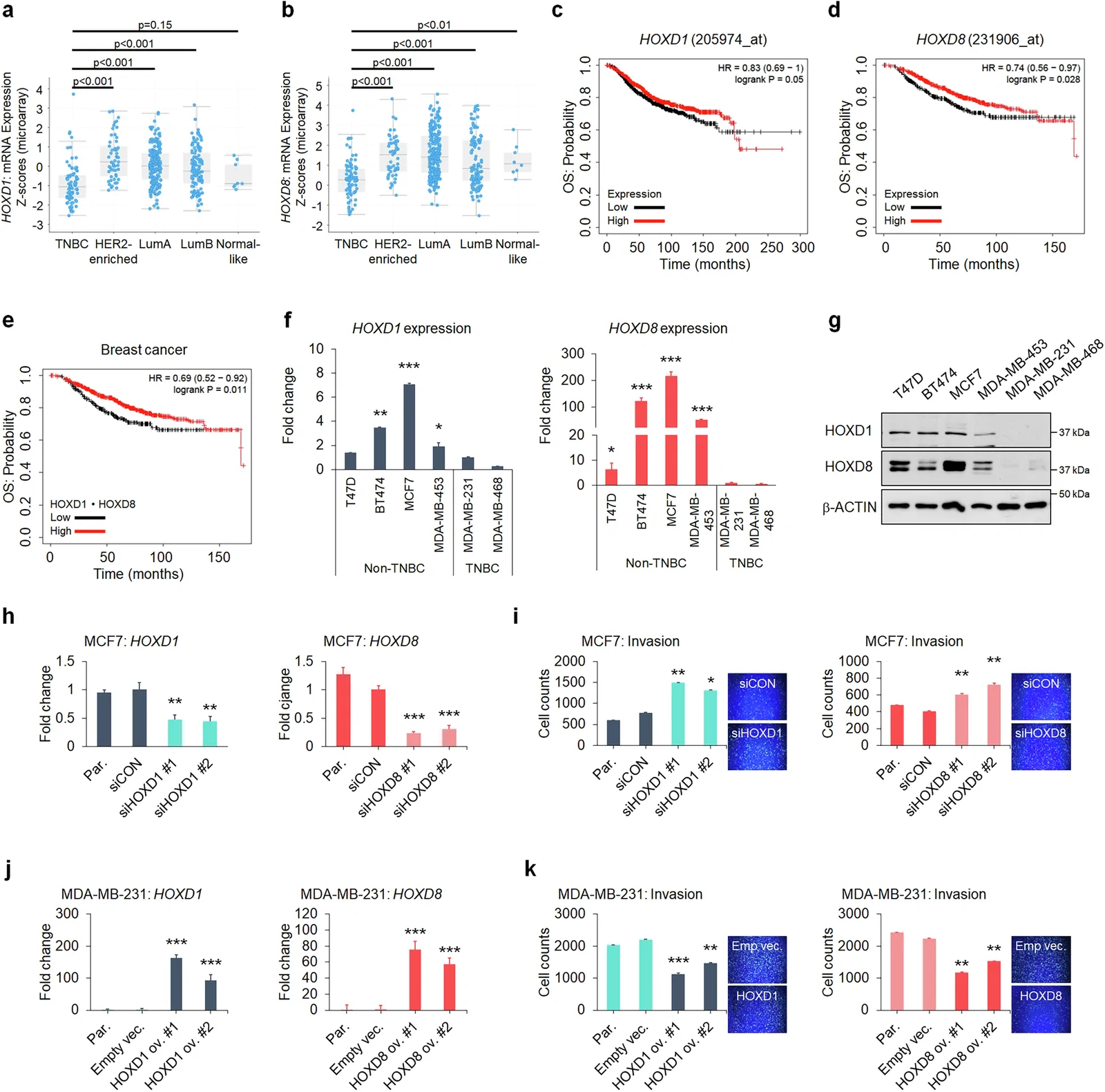
*HOXD1* and *HOXD8* downregulation correlates with TNBC. **a**, **b** Analyses of publicly available TCGA dataset of patients with BC showing the expression levels of *HOXD1* (**a**) and *HOXD8* (**b**) across different BC subtypes. **c**–**e** Kaplan–Meier OS analyses comparing high and low levels of *HOXD1* (**c**), *HOXD8* (**d**), or combined *HOXD1* and *HOXD8* expression (**e**) in BC. For survival analysis, the *HOXD1* (2055974_at) and *HOXD8* (231906_at) probes were selected and analyzed. **f** Detection of *HOXD1* and *HOXD8* mRNA expression levels in different human BC cell lines by RT-qPCR. Relative expression levels were normalized to MDA-MB-231 cells. **g** Lysates from different human BC cell lines were subjected to western blotting to examine HOXD1 and HOXD8 protein levels. **h** Expression levels of perturbed *HOXD1* (left panel) and *HOXD8* (right panel) in MCF7 cells. **i** Transwell assays of MCF7 cells transfected with siCON or siHOXD1 (left panel) and HOXD8 (right panel). **j** Expression levels of overexpressed *HOXD1* (left panel) and *HOXD8* (right panel) in MDA-MB-231 BC cells. **k** Transwell assays of MDA-MB-231 cells transfected with Empty vec., HOXD1 ov. (left panel), or HOXD8 ov. (right panel). Data represent mean ± s.e.m. from three independent biological replicates. Statistical significance was determined using Student’s *t*-test. *P < 0.05, **P < 0.01, ***P < 0.001.

To determine whether HOXD family members exhibit subtype-associated expression patterns in BC, we analyzed HOXD gene expression profiles using TCGA datasets. Among the HOXD family members, *HOXD1* and *HOXD8* showed the most consistent subtype-specific downregulation in patients with TNBC compared with other BC subtypes. (Fig. 1a, b), whereas other HOXD cluster genes (*HOXD3, D4, D9, D10, D11, D12, and D13*) did not exhibit statistically significant subtype-specific expression patterns (Supplementary Fig. 1). Kaplan–Meier survival analysis revealed that lower individual expression of *HOXD1* and *HOXD8* was significantly associated with poor overall survival (OS) in patients with BC (Fig. 1c,d). In addition, combining the low expression of both genes accentuated the poor OS curve (Fig. 1e). Notably, correlation analyses using the CCLE database revealed that *HOXD1* and *HOXD8* were the top mutually correlated genes among BC cell lines (Supplementary Fig. 2), further supporting the possibility of coordinated regulation between the two genes. Collectively, these findings support the subtype-specific downregulation and coordinated expression of *HOXD1* and *HOXD8* in TNBC.

We validated these findings in vitro by comparing relative expression levels of *HOXD1* and *HOXD8* between non-TNBC (e.g., MCF7, BT474) and TNBC (e.g., MDA-MB-231) cell lines. RT-qPCR and western blot analyses consistently demonstrated lower *HOXD1* and *HOXD8* expression in TNBC cells compared with non-TNBC cells (Fig. 1f, g), consistent with the in silico dataset analyses (Fig. 1a, b).

To elucidate the functional role of *HOXD1* and *HOXD8* in BC progression, we performed loss-of-function studies in non-TNBC cell lines (MCF7 and BT474). The knockdown of *HOXD1* and *HOXD8* using siRNAs significantly enhanced the invasive and migratory capabilities of MCF7 cells (Fig. 1h, i and Supplementary Fig. 3a, b). Similar increases in invasion and migration were observed in HOXD1- and HOXD8-perturbed BT474 cells compared with control cells (Supplementary Fig. 3c, d). In contrast, overexpression of *HOXD1* and *HOXD8* in MDA-MB-231 TNBC cells resulted in the opposite effect—a significant suppression of invasion and migration (Fig. 1j, k and Supplementary Fig. 3e, f). In addition, MTT-based proliferation assays demonstrated that depletion of *HOXD1* or *HOXD8* increased the proliferation of MCF7 cells (Supplementary Fig. 3g), whereas overexpression of *HOXD1* or *HOXD8* reduced the proliferation of MDA-MB-231 cells (Supplementary Fig. 3h). Together, these results indicate that specific downregulation of *HOXD1* and *HOXD8* is associated with TNBC-associated aggressive phenotypes.

### Distinct genomic architecture of the HOXD cluster in non-TNBC and TNBC cells

**Fig. 2:**
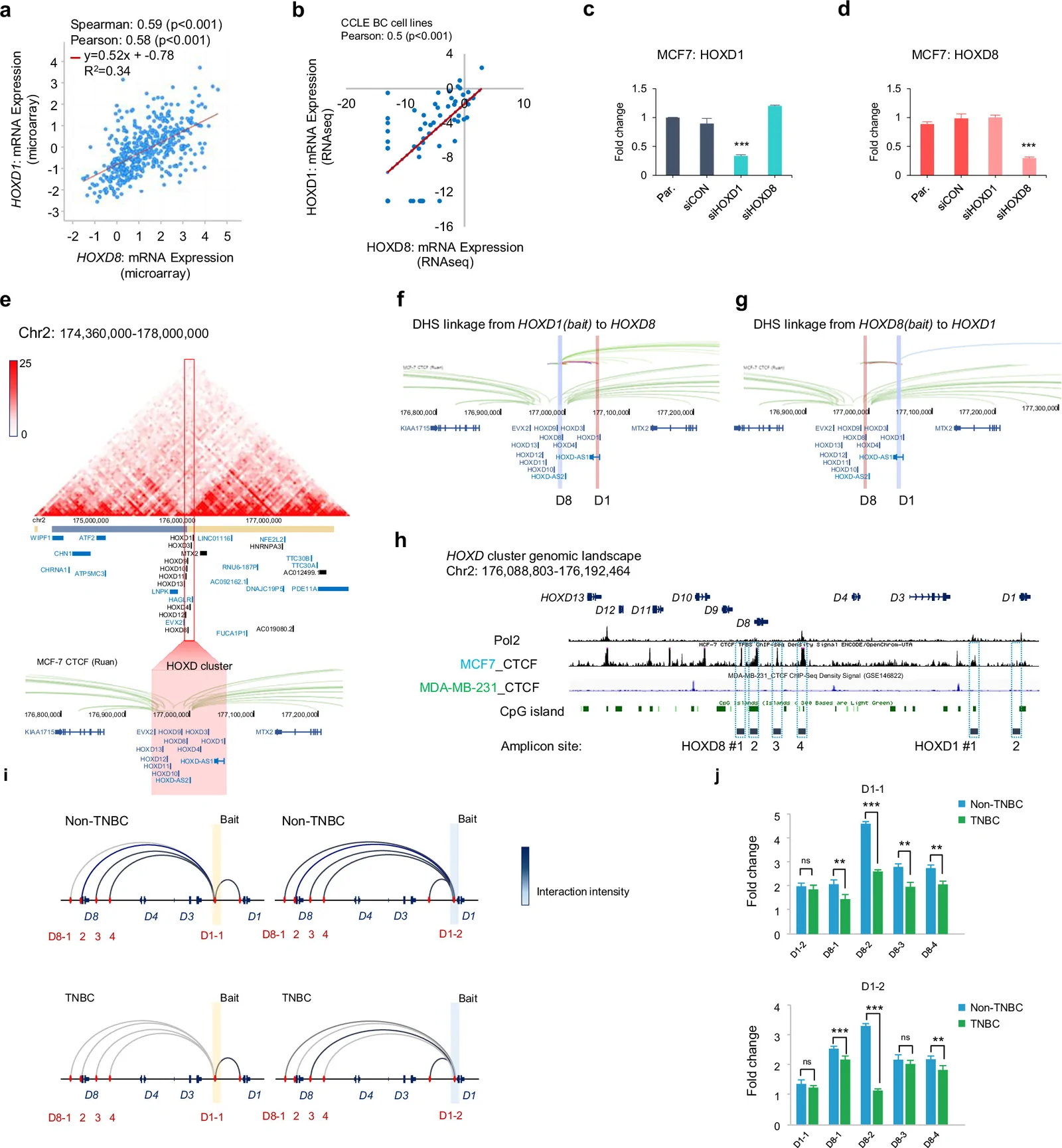
Distinct genomic architecture of the HOXD cluster between non-TNBC and TNBC cells. **a**, **b** Correlation curve between *HOXD1* and *HOXD8* expression in TCGA BC patient tissues (**a**) and CCLE BC cell lines (**b**). **c**, **d** RT-qPCR of *HOXD1* (**c**) and *HOXD8* (**d**) in MCF7 cells transfected with siHOXD1 or siHOXD8. Statistical analyses in **c** and **d** were performed relative to the siCON group. **e** Topologically associated domains (TADs) within the HOXD cluster in MCF7 cells obtained from UCSC Genome Browser datasets (upper panel). Visualized chromatin interactions obtained from CTCF–ChIA–PET analysis (lower panel). **f** Visualized 4C data presenting chromatin linkage from *HOXD1* to *HOXD8*. **g** Visualized 4C data presenting chromatin linkage from *HOXD8* to *HOXD1*. **h** UCSC Genome Browser view of the HOXD locus (on chr2:176,088,803-176,192,464) showing RNA polymerase II (Pol2), CTCF ChIP-seq, and CpG island tracks from ENCODE and GSE146822 dataset resources. Potential CBSs are indicated by dark blue bars below the corresponding CTCF peak signals. **i** Visualized schematic representation of CTCF-associated chromatin interactions within the HOXD cluster in non-TNBC and TNBC cells. Color intensity represents relative chromatin interaction frequencies detected by 3C analysis. 3C–PCR analyses were performed using D1-1 (left panels) or D1-2 (middle panels) as bait sites against the indicated HOXD8-associated CBSs in non-TNBC (upper panels) and TNBC (lower panels) cells. **j** Relative chromatin interaction frequencies are shown in the bar graphs. Statistical significance was determined using Student’s *t*-test. *P < 0.05, **P < 0.01, ***P < 0.001. *ns* not significant.

Although HOX genes are typically regulated as a cluster exhibiting collinear expression^26^, analysis of TCGA and CCLE datasets for BC showed a specific positive correlation only between *HOXD1* and *HOXD8*, excluding other HOXD cluster genes (Fig. 2a, b, Supplementary Fig. 2). Furthermore, cross-regulation experiments confirmed that *HOXD1* and *HOXD8* do not regulate each other’s expression (Fig. 2c, d), suggesting that their coordinated expression may instead be influenced by shared upstream regulatory mechanisms.

To elucidate the regulatory mechanism underlying the coordinated regulation of non-adjacent *HOXD1* and *HOXD8* genes despite the presence of intermediate HOXD genes, we hypothesized that chromatin interactions associated with active and inactive chromatin states may contribute to their co-regulation. Therefore, we examined the spatial organization of the HOXD locus in MCF7 cells, noting that the HOXD cluster is positioned near the border of a subtopologically associating domain (subTAD) (Fig. 2e). ChIA–PET data generated using CTCF immunoprecipitation revealed partitioned chromatin interaction patterns extending in two directions across the HOXD locus, suggesting a potential role of CTCF-associated chromatin organization at this subTAD boundary region (Fig. 2e). Circularized chromosome conformation capture (4C) data further supported chromatin interaction patterns between *HOXD1* and *HOXD8* when either was set as the bait site (Fig. 2f, g). These results suggest that CTCF-associated chromatin interactions may contribute to the spatial organization of the *HOXD1* and *HOXD8* loci.

As the ability of CTCF to mediate chromatin interactions is dependent on DNA binding^27^, CTCF enrichment status was investigated using publicly available UCSC Genome Browser/ENCODE datasets. CTCF ChIP-seq analysis in MCF7 cells displayed two CBSs near *HOXD1* and four CBSs near the *HOXD8* locus, whereas CTCF ChIP-seq analysis in MDA-MB-231 cells (GES146822) revealed reduced CTCF occupancy at the same CBSs (Fig. 2h). To further investigate chromatin interactions between *HOXD1* and *HOXD8*, we performed 3C analysis in both non-TNBC and TNBC cells. Chromatin interactions between *HOXD1* and *HOXD8* were more prominently detected in MCF7 cells than in MDA-MB-231 cells, which exhibited lower *HOXD1* and *HOXD8* expression levels (Fig. 2i, j). This suggests a potential association of CTCF occupancy with subtype-specific chromatin interaction patterns at the *HOXD1* and *HOXD8* loci.

### Altered CTCF occupancy is associated with chromatin changes between *HOXD1* and *HOXD8* in BCs

**Fig. 3:**
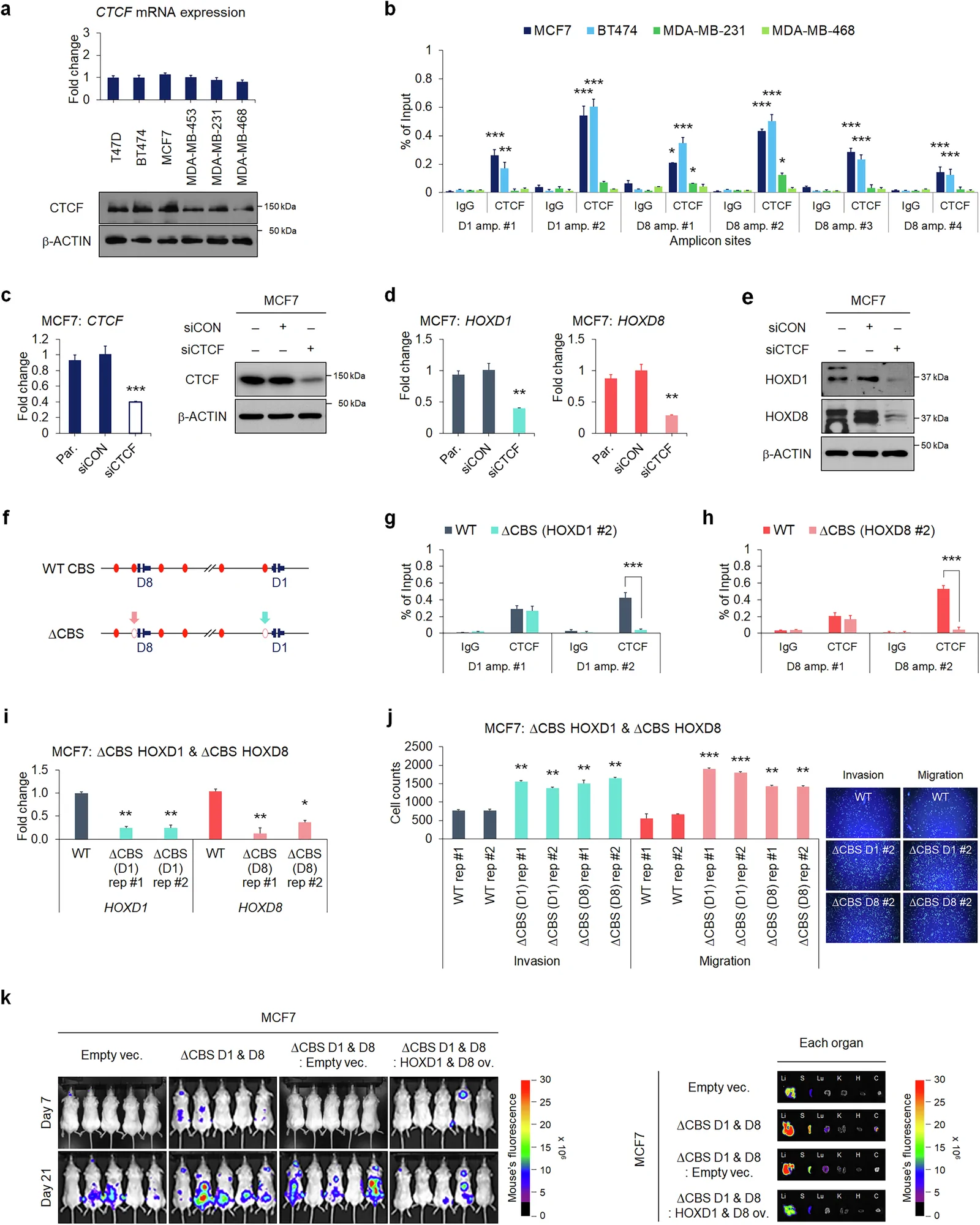
CTCF-associated chromatin changes between HOXD1 and HOXD8 in BC cells. **a** Detection of CTCF mRNA (upper panel) and protein (bottom panel) expression in different human BC cell lines. **b** ChIP analysis of CTCF enrichment at the *HOXD1* and *HOXD8* promoter regions in non-TNBC and TNBC cell lines. IgG was used as a negative control. **c** mRNA (left panel) and protein (right panel) levels of CTCF knockdown in MCF7 cells. **d**, **e** mRNA (**d**) and protein (**e**) levels of *HOXD1* and *HOXD8* in CTCF-depleted MCF7 cells. **f** Schematic representation of CBSs located near *HOXD1* and *HOXD8*. Deletion of specific CBSs (HOXD1 #2 and HOXD8 #2) using the CRISPR–Cas9 system is indicated by empty red circles. **g**, **h** ChIP analysis of CTCF enrichment status at the HOXD1 #2 (**g**) and HOXD8 #2 (**h**) regions in CBS-deleted MCF7 cells. **i** Expression levels of downregulated *HOXD1* and *HOXD8* in CBS-deleted MCF7 cells. **j** Transwell assays of CBS-deleted MCF7 cells. k Representative IVIS images of xenograft mouse models injected with MCF7: Empty vec., MCF7: △CBS D1& D8, and MCF7: △CBS D1 & D8: HOXD1 & D8 ov. cells at the indicated time points (left). Tumor volumes were measured at the indicated time points. Representative ex vivo fluorescence images of the harvested liver (Li), spleen (S), lung (Lu), kidney (K), heart (H), and primary cancer tissues (C) from xenograft mice (right). Statistical significance was determined using one-way ANOVA. *P < 0.05, **P < 0.01, ***P < 0.001.

To investigate whether differential HOXD1/HOXD8 regulation was associated with altered CTCF expression, we first examined total CTCF mRNA and protein levels in non-TNBC and TNBC cells. No significant differences in overall CTCF expression were observed between the two subtypes (Fig. 3a). Next, to examine whether CTCF occupancy at the HOXD locus differs according to BC subtype, ChIP assays were performed at several candidate amplicon sites (Fig. 3b). This revealed that the extent of binding at the *HOXD1* and *HOXD8* loci was strongly enriched in non-TNBC cells compared with TNBC cells, suggesting a difference in the binding strength or accessibility depending on the subtype (Fig. 3b).

To assess the functional relevance of CTCF in *HOXD1* and *HOXD8* regulation, we knocked down CTCF in MCF7 cells using siRNAs (Fig. 3c). CTCF knockdown significantly diminished the expression of both *HOXD1* and *HOXD8* compared with parent and control siRNA-transfected MCF7 cells (Fig. 3d, e). Next, specific CBSs (ΔCBS) located near the putative regulatory regions of HOXD1 and HOXD8 were deleted using the CRISPR–Cas9 system (Fig. 3f). MCF7 ΔCBS cells showed decreased CTCF enrichment (Fig. 3g,h), and a corresponding reduction in *HOXD1* and *HOXD8* gene expression (Fig. 3i), which in turn enhanced aggressive cellular phenotypes (Fig. 3j). Consistent with these findings, xenograft tumors derived from ΔCBS cells exhibited enhanced tumor progression, whereas re-expression of *HOXD1* and *HOXD8* attenuated these in vivo phenotypes (Fig. 3k). Collectively, these findings support an association between CTCF occupancy, chromatin interaction patterns, and coordinated regulation of *HOXD1* and *HOXD8* in BC cells.

To further investigate molecular alterations associated with the observed in vivo phenotypes, xenograft tumor tissues were analyzed by RT-qPCR (Supplementary Fig. 4). Tumors derived from ΔCBS D1 & D8 cells exhibited reduced *HOXD1* and *HOXD8* expression, with increased proliferation- and invasion/EMT-associated gene expression, whereas re-expression of *HOXD1* and *HOXD8* largely reversed these changes (Supplementary Fig. 4a–c). Similarly, xenograft tumors generated from MDA-MB-231 cells overexpressing HOXD1 and HOXD8 showed reduced tumor progression-associated molecular signatures (Supplementary Fig. 4d, e). These findings further support the association between CTCF-associated chromatin interactions and HOXD1/HOXD8 regulation in BC cells.

### DNA demethylation is linked to altered CTCF occupancy at the HOXD locus in BC cells

**Fig. 4:**
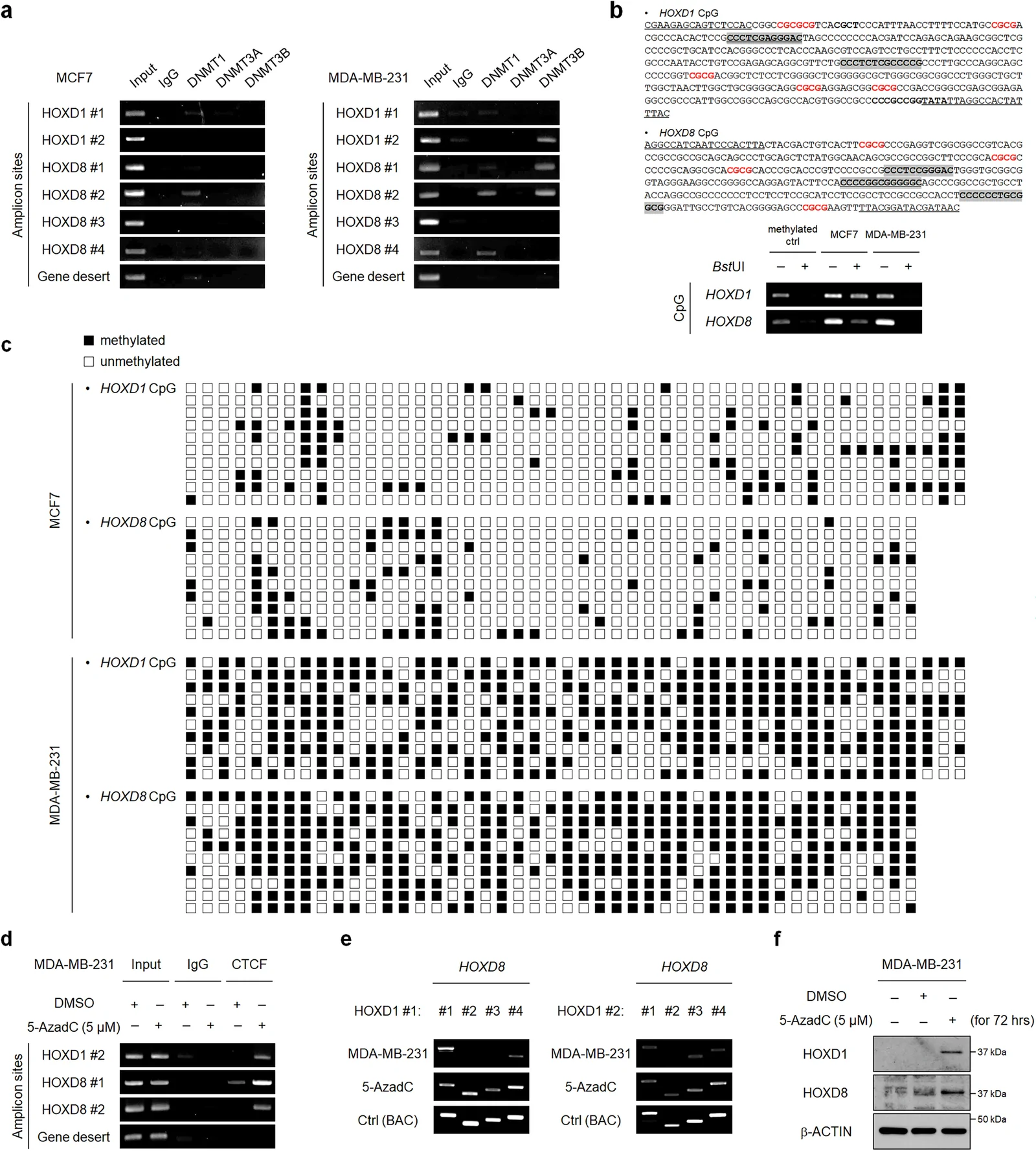
DNA demethylation by 5-AzadC treatment is associated with altered CTCF occupancy at the HOXD locus in BC cells. **a** ChIP analysis of DNMT1, DNMT3A, and DNMT3B enrichment at regions near the *HOXD1* and *HOXD8* loci in non-TNBC and TNBC cell lines. IgG was used as a negative control. **b** Genomic DNA sequences of the analyzed amplicons: primer sequences are underlined, and the BstUI recognition sequences (CGCG) used for COBRA are indicated in red (upper panel). COBRA analysis using genomic DNA derived from MCF7 and MDA-MB-231 cells (lower panel). **c** Bisulfite sequencing analysis of the amplicon generated from MCF7 and MDA-MB-231 cells. Methylated CpGs are represented by black squares and unmethylated sites by open squares. **d** CTCF-binding analysis at the HOXD1 #2, HOXD8 #1, and HOXD8 #2 sites through ChIP–PCR using an antibody against CTCF in 5-AzadC-treated and non-treated MDA-MB-231 cells. **e** Representative 3C–PCR analysis of three replicate experiments performed in both MDA-MB-231 and 5-AzadC-treated MDA-MB-231 cells. A BAC clone containing the HOXD locus was digested, ligated, and used as the 3C control template. **f** Increased protein expression levels of HOXD1 and HOXD8 in 5-AzadC-treated MDA-MB-231 cells compared with control cells. Data represent mean ± s.e.m. from three independent biological replicates. Statistical significance was determined using Student’s *t*-test (*P < 0.05, **P < 0.01, ***P < 0.001).

It has been previously reported that CTCF binding is often sensitive to DNA methylation at the CpG dinucleotides within its core binding sequence^27^. As the DNA methylation status is regulated by the DNA methyltransferase (DNMT) family, including DNMT1, DNMT3a, and DNMT3b, we investigated whether DNMT accumulation was associated with differential regulation at the *HOXD1*/*HOXD8* loci. ChIP assay revealed that DNMT3b was generally more accumulated in TNBC cells than in non-TNBC cells at the HOXD1 and HOXD8 amplicon sites (Fig. 4a). We then determined the methylation status using COBRA and bisulfite sequencing to determine whether the accumulation of DNMTs at the HOXD1/HOXD8 amplicon sites indeed led to hypermethylation. The non-TNBC cell line MCF7 displayed a hypomethylation pattern, whereas the TNBC cell line MDA-MB-231 showed a distinct hypermethylation status at the putative promoter regions of *HOXD1*/*HOXD8* (Fig. 4b, c).

Given that DNA methylation at CBSs has been linked to reduced CTCF occupancy, we next investigated whether demethylation could influence *HOXD1*/*HOXD8* gene activation in TNBC cells. MDA-MB-231 cells were treated with 5-AzadC, a DNMT inhibitor^28^, and the effects on CTCF occupancy and *HOXD1*/*HOXD8* expression were examined. ChIP–PCR analysis revealed that 5-AzadC treatment enhanced CTCF enrichment at the *HOXD1*/*HOXD8-*binding regions (Fig. 4d). We next examined whether demethylation was associated with altered chromatin interaction patterns at the HOXD locus using 3C analysis in 5-AzadC-treated MDA-MB-231 cells. Interestingly, demethylation partially altered chromatin interaction patterns within the HOXD cluster (Fig. 4e**)**, accompanied by increased *HOXD1* and *HOXD8* expression in 5-AzadC-treated MDA-MB-231 cells (Fig. 4f**)**. Together, these findings support an association between DNA methylation status, CTCF occupancy, chromatin interaction patterns, and *HOXD1*/*HOXD8* expression at the HOXD locus.

### HOXD1 and HOXD8 regulate TNBC progression through *FOXA1*

**Fig. 5:**
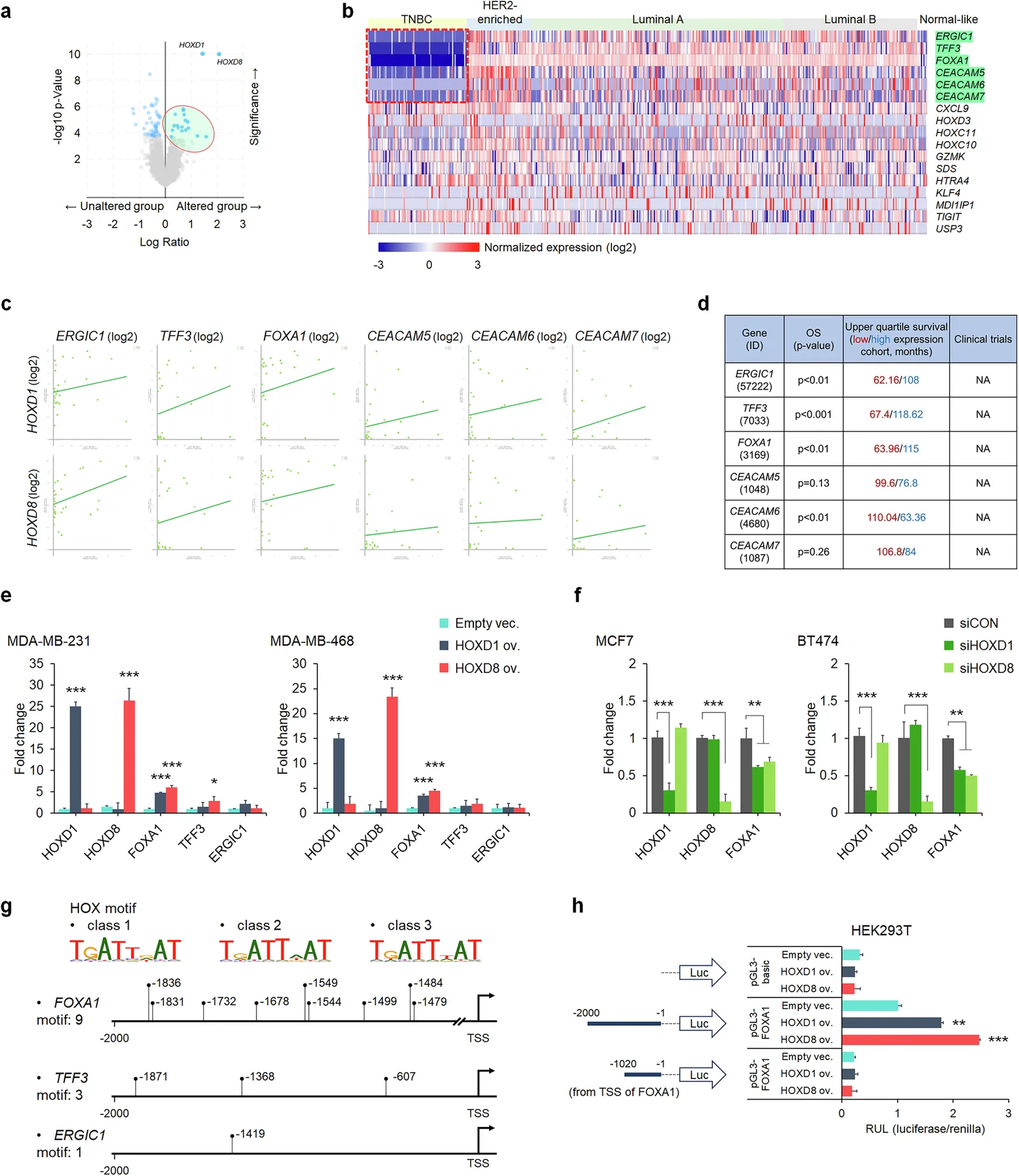
*FOXA1* is a potential downstream transcriptional target associated with HOXD1 and HOXD8 in TNBC. **a** Volcano plot showing differential gene expression analysis between *HOXD1*/*HOXD8*-altered and unaltered BC tissues from the TCGA dataset (GSE65194). Blue dots indicate significantly differentially expressed genes associated with *HOXD1*/*HOXD8* alteration. **b** Heatmap showing normalized expression fold changes of candidate *HOXD1*/*HOXD8*-associated genes across patient samples with BC. Each column represents an individual patient. **c** Correlation analysis between *HOXD1*/*HOXD8* expression and TNBC-associated downregulated genes (*ERGIC1, TFF3, FOXA1, CEACAM5, CEACAM6*, and *CEACAM7*). **d** Kaplan–Meier OS analyses comparing high and low levels of potential *HOXD1/HOXD8* downstream target genes in BC. For survival analysis, the *ERGIC* (2055974_at), *TFF3* (204623_at), *FOXA1* (204667_at), *CEACAM5* (217291_at), *CEACAM6* (203757_at), and *CEACAM7* (206199_at) probes were analyzed. **e** Expression levels of *ERGIC*, *TFF3*, and *FOXA1* genes in *HOXD1/HOXD8* overexpressing MDA-MB-231 (left panel) and MDA-MB-468 (right panel). **f** Expression levels of *FOXA1* in *HOXD1/HOXD8*-depleted MCF7 cells (left panel) and BT474 (right panel) cells. **g** Three classes of HOX consensus sequences (upper panel) and the distribution of predicted HOX consensus motifs within the promoter regions of *ERGIC1, TFF3*, and *FOXA1* (lower panel). **h** Schematic illustration of the *FOXA1* promoter regions cloned into the pGL3 luciferase reporter plasmid (left panel). Quantification of the luciferase activity of the *FOXA1* promoter reporter was examined in HEK293T cells (right panel). Luciferase activities were measured from three independent experiments performed in triplicate. Data represent mean ± s.e.m. from three independent biological replicates. Statistical significance was determined using Student’s *t*-test. *P < 0.05, **P < 0.01, ***P < 0.001).

To identify potential downstream transcriptional targets of *HOXD1* and *HOXD8*, we performed an in silico correlation analysis of genes showing coordinated expression patterns with *HOXD1* and *HOXD8* (Fig. 5a). Using a twofold change cut-off, 17 co-dysregulated genes were identified (Supplementary Fig. 5). A heatmap analysis of their expression across different BC subtypes narrowed the focus to six genes (*ERGIC1, TFF3, FOXA1, CEACAM5, CEACAM6*, and *CEACAM7*) showing differential expression between TNBC and other subtypes (Fig. 5b), which positively correlated with *HOXD1*/*HOXD8* expression in various BC cell lines (Fig. 5c). Of these genes, low expression of *ERGIC1*, *TFF3*, and *FOXA1* was strongly correlated with poor OS in patients with BC (Fig. 5d and Supplementary Fig. 6).

RT-qPCR was used to validate the transcription levels of *ERGIC1*, *TFF3*, and *FOXA1* in *HOXD1*- or *HOXD8*-overexpressing TNBC cell lines. *FOXA1* was the most significantly upregulated gene upon *HOXD1* or *HOXD8* overexpression (Fig. 5e). Conversely, *FOXA1* was notably downregulated when *HOXD1* or *HOXD8* were knocked down in non-TNBC cell lines (MCF7 and BT474 cells) (Fig. 5f). In addition, bioinformatic analysis identified nine HOX consensus motifs within the promoter region of *FOXA1* (Fig. 5g). Furthermore, to investigate whether *HOXD1* and *HOXD8* function as transcriptional regulators of *FOXA1*, a luciferase promoter assay was performed. Overexpression of *HOXD1* or *HOXD8* significantly increased *FOXA1* promoter activity in the −2000 to −1 promoter construct, whereas the −1020 to −1 construct did not show a comparable response (Fig. 5h). These findings suggest that regulatory elements located between −2000 and −1020, which contain multiple predicted HOX consensus motifs, may contribute to *FOXA1* transcriptional activation. Together, these data suggest that *FOXA1* may represent a potential downstream transcriptional target regulated by *HOXD1* and *HOXD8*. Consistent with these findings, xenograft tumor tissues derived from MCF7 ΔCBS D1 & D8 cells exhibited reduced *FOXA1* expression, whereas re-expression of *HOXD1* and *HOXD8* restored *FOXA1* levels (Supplementary Fig. 4a). Similarly, *FOXA1* expression was increased in xenograft tumors generated from MDA-MB-231 cells overexpressing *HOXD1* and *HOXD8* (Supplementary Fig. 4e), further supporting an association between *HOXD1*/*HOXD8* expression and *FOXA1* regulation in BC cells.

## Discussion

Here, we identified a critical regulatory axis—CTCF/*HOXD1*/*HOXD8*/*FOXA1*—that is suppressed in TNBC. We demonstrated that the transcriptional levels of *HOXD1* and *HOXD8* are uniquely lower in TNBC than in other BC subtypes, and that this downregulation is associated with poor prognosis. In non-TNBC cells, CTCF binds to hypomethylated CpG-rich regions near *HOXD1*/*HOXD8*, contributing to CTCF-associated chromatin interactions correlated with active gene expression. Conversely, in TNBC, DNMT3b-associated promoter hypermethylation correlates with reduced CTCF occupancy and CTCF-associated chromatin changes, accompanied by decreased expression of *HOXD1* and *HOXD8*.

**Fig. 6:**
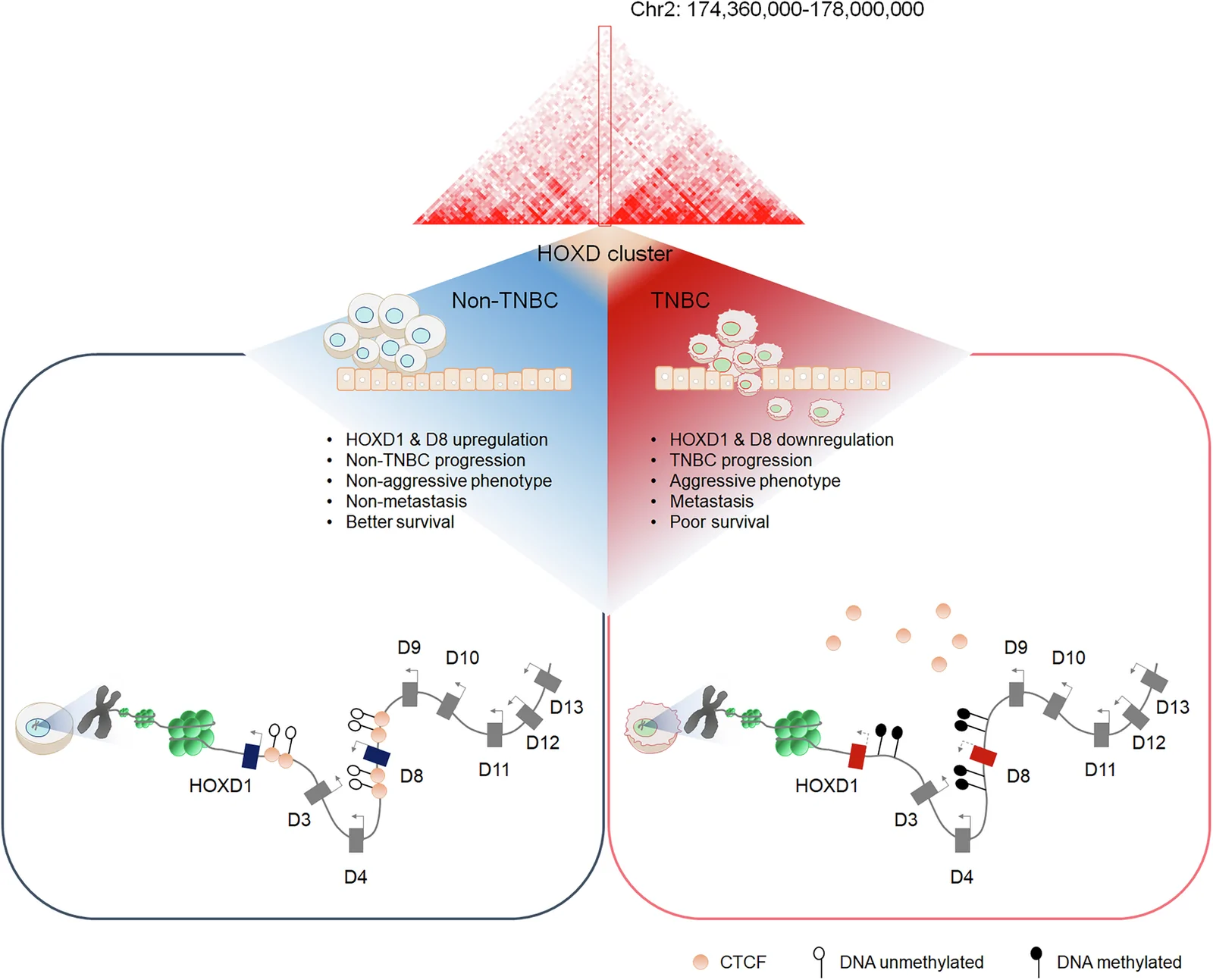
Proposed model of CTCF-associated epigenetic regulation of *HOXD1* and *HOXD8* in TNBC. Reduced *HOXD1* and *HOXD8* expression in TNBC was associated with aggressive tumor-associated phenotypes. Functional analyses demonstrated that depletion of *HOXD1*/*HOXD8* promoted invasion, migration, and proliferation, whereas overexpression attenuated these phenotypes. Coordinated regulation at the HOXD locus was associated with CTCF occupancy and DNA methylation status near the HOXD1 and HOXD8 regulatory regions. Compared with non-TNBC cells, TNBC cells exhibited promoter hypermethylation accompanied by reduced CTCF occupancy and CTCF-associated chromatin changes. Together, these observations suggest that epigenetic regulation and altered CTCF occupancy contribute to coordinated downregulation of HOXD1 and HOXD8, thereby promoting the aggressive phenotype of TNBC.

We demonstrated that disrupting the CBSs using CRISPR–Cas9 leads to the downregulation of *HOXD1* and *HOXD8* expression and their downstream target *FOXA1*, ultimately resulting in increased cancer aggression, progression, metastasis, and poor prognosis (Fig. 6). Importantly, forced expression of *HOXD1* and *HOXD8* in TNBC cells effectively suppresses aggressive phenotypes in vitro and tumor progression in vivo. These findings support a tumor-suppressive role for *HOXD1* and *HOXD8* in TNBC.

HOX genes are broadly implicated in various cancers, functioning as either oncogenes or tumor suppressors that regulate proliferation, cell death, angiogenesis, invasion, and metastasis^16^. Several studies have reported that *HOXA13*, *HOXB2*, *HOXB3*, *HOXC4*, and *HOXC6* are involved in signaling pathways related to sustained proliferation^17,29,30^. In addition, *HOXA9*, *HOXB2*, *HOXB5*, *HOXB9*, and *HOXC8* have been associated with the trait of resistance to cell death^17,31^. Consistent with these observations, we have also previously reported that *HOXB5* promotes tumor aggression and progression and confers resistance to endocrine therapy for patients with BC^32^. Among the HOX family members, the oncogenic role of HOXA9 has been most extensively characterized in acute myeloid leukemia (AML)^33,34^. Elevated HOXA9 expression is observed in a substantial proportion of AML cases and is strongly associated with poor clinical outcomes and an unfavorable prognosis^33,35^. Moreover, HOXA9 overexpression is both necessary and sufficient for leukemic transformation, highlighting its pivotal role as a master regulator of malignant progression^36–38^. Mechanistically, HOXA9 regulates numerous downstream targets involved in stem cell maintenance, proliferation, differentiation blockade, and cell survival. Its expression is controlled by multiple genetic and epigenetic mechanisms, including chromosomal rearrangements, transcriptional regulators, and higher-order chromatin organization^34,39^. Recent studies have further demonstrated that 3D chromatin interactions and epigenetic regulators contribute to the maintenance of aberrant HOXA9 expression in leukemia^39^. Therefore, the well-established example of HOXA9 dysregulation in AML supports the concept that spatial chromatin organization and context-dependent regulation of HOX genes represent important mechanisms underlying tumorigenesis^33,39^. In this regard, our findings suggest that the altered chromatin architecture and coordinated downregulation of HOXD1 and HOXD8 in TNBC represent another example of HOX gene dysregulation associated with cancer progression, thereby extending the biological significance of HOX gene regulation beyond hematologic malignancies.

These roles of HOX genes in cancer development reportedly often involve regulatory interactions with other molecular elements, such as lncRNAs and microRNAs^40^. For instance, we have shown that HOXA1 regulates endocrine resistance in ER+ BC cells by interacting with its neighboring lncRNA HOTAIRM1, which directly interacts with EZH2 and prevents the PRC2 complex from binding and depositing H3K27me3 at the putative promoter of HOXA1^41^. According to a previous study, the lncRNA HOXD-AS1, located between *HOXD1* and *HOXD3*, reportedly suppresses carcinoma growth and metastasis in colon cancer by inhibiting integrin β3 transcriptional activation and MAPK/AKT signaling^42^. Another report demonstrated the role of HOXD-AS1 in tumor progression and poor prognosis in colorectal cancer by competing with miR-217^43^. Although our study primarily focuses on the chromatin interaction between HOXD1 and HOXD8, further investigation of adjacent lncRNAs, including HOXD-AS1, and associated signaling pathways may provide a more comprehensive understanding of the molecular mechanisms by which HOXD1 and HOXD8 contribute to TNBC progression.

CTCF has been identified as an important driving factor in various cancers, including BC^44,45^. CTCF has multifunctional properties as a transcriptional activator, repressor, and insulator through regulation of 3D chromatin structure^46^. Disruption of specific CBSs can represent a tissue-specific mechanism of tumorigenesis, as shown in previous studies where mutations at CBSs were reportedly associated with gastric and colon carcinogenesis^47^. In addition, the absence of CTCF binding to the target site altered the expression levels of cell proliferation-related genes such as *MYC*, *p19ARF*, and *Igf2*^48^. A role for CTCF as a regulator of chromatin looping has been shown by studies demonstrating altered connectivity at distal and proximal estrogen receptors at the P2RY2 locus, with reduced CTCF binding after ER treatment^49^. In this study, using in silico dataset analysis, we observed that the HOXD comprises identical subTADs in non-TNBC cells, suggesting a high degree of chromatin interactions within this cluster. We showed that CTCF binding mediates distinct chromatin interactions at the HOXD locus between non-TNBC and TNBC, correlating with the downregulation of *HOXD1* and *HOXD8*. Interestingly, although HOXD1 and HOXD8 are not physically adjacent within the HOXD cluster, both genes exhibited coordinated downregulation in TNBC, whereas the intermediate HOXD family members remained relatively unaffected. One possible explanation is that the CTCF-mediated subTAD architecture may facilitate selective long-range enhancer–promoter interactions that spatially connect HOXD1 and HOXD8 while bypassing the intermediate genes^22,50,51^. Such chromatin loops could establish a functional regulatory unit in which disruption of CTCF occupancy preferentially affects the expression of specific target genes despite their linear genomic distance^22,52^. Alternatively, the intervening HOXD genes may be regulated by distinct promoters, enhancers, or epigenetic mechanisms that render them less dependent on the CTCF-associated chromatin interactions identified in this study^53,54^. Therefore, the spatial selectivity observed within the HOXD subTAD suggests that chromatin organization contributes to gene-specific regulation within the HOXD cluster^50,51,55^. As CTCF can modulate gene expression by reorganizing chromatin interactions with enhancers and other transcription factors, future studies integrating chromatin interaction analyses, enhancer mapping, and epigenetic profiling will be required to fully delineate the regulatory mechanisms underlying the selective downregulation of HOXD1 and HOXD8 in TNBC^22,51,55^.

To our knowledge, this study is the first to identify coordinated, subtype-specific downregulation of HOXD1 and HOXD8 in TNBC associated with altered CTCF occupancy and DNA methylation at the HOXD locus. Our findings further suggest that these epigenetic alterations are linked to CTCF-associated chromatin changes, thereby contributing to reduced HOXD1/HOXD8 expression and the aggressive phenotype of TNBC. In addition, we identified *FOXA1* as a potential downstream transcriptional target associated with *HOXD1*/*HOXD8* signaling. Collectively, these findings provide insight into the interplay between DNA methylation, CTCF occupancy, and HOXD cluster gene regulation in TNBC, and suggest that this regulatory axis represents a potential therapeutic target in aggressive BC.

## Supplementary information

Supplementary Information

## Data Availability

The datasets generated during the current study, including molecular assays, chromatin interaction analyses, CRISPR-edited cell line data, and in vivo xenograft data, are available from the corresponding author upon reasonable request. Publicly available datasets used in this study, including TCGA, GEO, CCLE, and Kaplan–Meier plotter datasets, are described in the Methods section and cited appropriately in the manuscript.
