# Supplementary material for: CTCF-associated chromatin changes between HOXD1 and HOXD8 contribute to TNBC progression
Source: Exp Mol Med. 2026 Oct 6;58(10):3486–98. doi: 10.1038/s12276-026-01861-6 (PMC13737077; doi:10.1038/s12276-026-01861-6)
Supplement: Supplementary file 1 [file 12276_2026_1861_MOESM1_ESM.pdf]

Supplementary Figures

CTCF-associated chromatin changes between *HOXD1* and *HOXD8* contribute to TNBC progression

Ji Hoon Oh[1,†], Da Som Jeong[2,3], and Clara Yuri Kim[4]

[1] *Department of Biological Sciences, Keimyung University College of Natural Sciences, Daegu 42601, Republic of Korea*

[2] *Department of Internal Medicine, Yonsei University College of Medicine, Seoul 03722, Republic of Korea*

[3] *Yonsei Institute for Digital Health, Yonsei University, Seoul 03722, Republic of Korea*

[4] *Department of Genetics, Perelman School of Medicine, University of Pennsylvania, Philadelphia, PA, USA*

† Corresponding authors

Ji Hoon Oh (E-mail: jhoh@kmu.ac.kr Tel: +82-2-580-5208)

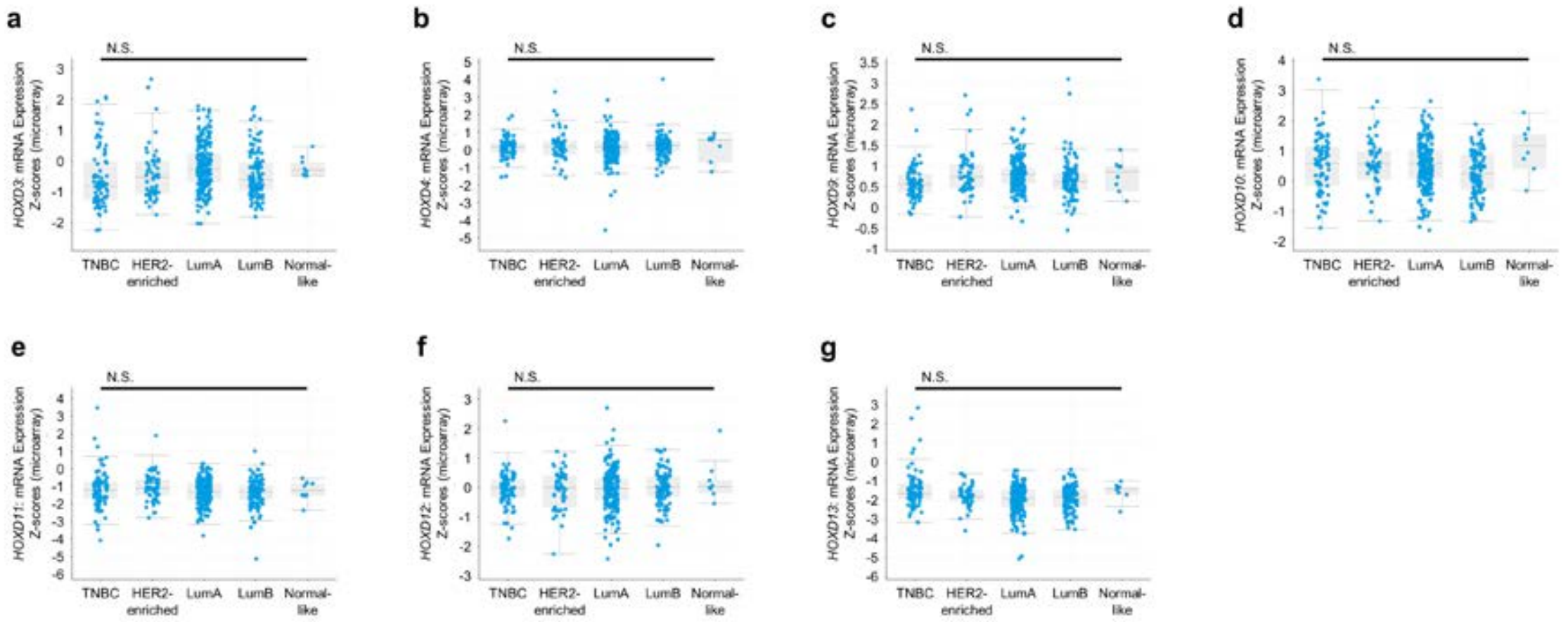

Supplementary Fig. 1. Expression levels of HOXD cluster genes in different subtypes of breast cancer. a-g, Analysis of TCGA dataset (Nature 2012) for the expression of *HOXD1* (**a**), *HOXD4* (**b**), *HOXD9* (**c**), *HOXD10* (**d**), *HOXD11* (**e**), *HOXD12* (**f**), and *HOXD13* (**g**) in different breast cancer subtypes.

Correlated gene of HOXD1

Correlated Gene	Cytoband	Spearman's Correlation	p-Value ▲	q-Value
HOXD8	2q31.1	0.593	4.77e-45	8.23e-41
SFRP4	7p14.1	0.482	3.57e-28	3.08e-24
ZCCHC24	10q22.3	0.478	1.24e-27	7.15e-24
CFH	1q31.3	0.477	1.71e-27	7.35e-24
CCDC80	3q13.2	0.474	3.57e-27	1.06e-23
CNRIP1	2p14	0.474	3.67e-27	1.06e-23
CFHR3	1q31.3	0.467	3.01e-26	7.42e-23
ZNF423	16q12.1	0.463	7.96e-26	1.72e-22
SFRP2	4q31.3	0.458	3.21e-25	6.15e-22
SERPINF1	17p13.3	0.456	4.91e-25	8.47e-22
KCNJ12	17p11.2	0.456	5.58e-25	8.74e-22
DCN	12q21.33	0.454	9.21e-25	1.32e-21
DACT1	14q23.1	0.449	2.95e-24	3.65e-21
FBN1	15q21.1	0.449	2.96e-24	3.65e-21
ECM2	9q22.31	0.449	3.62e-24	4.16e-21
TSHZ3	19q12	0.447	6.28e-24	6.77e-21
COL6A3	2q37.3	0.446	7.79e-24	7.90e-21
FAP	2q24.2	0.445	8.44e-24	8.08e-21
POSTN	13q13.3	0.445	9.84e-24	8.93e-21
SVEP1	9q31.3	0.442	2.04e-23	1.76e-20
GLT8D2	12q23.3	0.442	2.17e-23	1.79e-20
RUNX1T1	8q21.3	0.440	3.63e-23	2.72e-20
LOXL1	15q24.1	0.440	3.73e-23	2.72e-20
ITGBL1	13q33.1	0.439	3.79e-23	2.72e-20
CDH11	16q21	0.439	4.81e-23	3.32e-20

Showing 1-25 of 17236 ‹ Show more ›

Correlated gene of HOXD8

Correlated Gene	Cytoband	Spearman's Correlation	p-Value	q-Value ▲
HOXD1	2q31.1	0.593	4.77e-45	8.23e-41
JAM3	11q25	0.562	1.16e-39	1.00e-35
NDN	15q11.2	0.556	1.04e-38	5.98e-35
COL6A3	2q37.3	0.549	1.62e-37	6.97e-34
RUNX1T1	8q21.3	0.548	2.26e-37	7.77e-34
TCF4	18q21.2	0.541	2.64e-36	7.57e-33
CCDC80	3q13.2	0.540	3.14e-36	7.74e-33
GLT8D2	12q23.3	0.532	5.53e-35	1.19e-31
CNRIP1	2p14	0.532	6.66e-35	1.22e-31
BNC2	9p22.3-p22.2	0.531	7.07e-35	1.22e-31
SVEP1	9q31.3	0.530	1.19e-34	1.86e-31
ZCCHC24	10q22.3	0.527	2.77e-34	3.98e-31
LTBP2	14q24.3	0.523	1.03e-33	1.36e-30
ECM2	9q22.31	0.521	1.98e-33	2.41e-30
HTRA1	10q26.13	0.521	2.10e-33	2.41e-30
LAMA2	6q22.33	0.519	3.74e-33	4.02e-30
SFRP2	4q31.3	0.519	4.06e-33	4.12e-30
NID1	1q42.3	0.519	4.76e-33	4.55e-30
DCN	12q21.33	0.516	1.16e-32	1.05e-29
CFHR3	1q31.3	0.515	1.80e-32	1.48e-29
LRRC32	11q13.5	0.515	1.81e-32	1.48e-29
TSHZ3	19q12	0.513	2.85e-32	2.23e-29
CDH11	16q21	0.511	5.76e-32	4.32e-29
KERA	12q21.33	0.511	6.56e-32	4.71e-29
FLRT2	14q31.3	0.509	1.04e-31	6.92e-29

Showing 1-25 of 17236 ‹ Show more ›

Supplementary Fig. 2. Correlation analyses between HOXD1 and HOXD8 in breast cancer cell lines. Correlation analyses of genes associated with *HOXD1* and *HOXD8* expression in breast cancer cell lines using the CCLE database. *HOXD8* was identified as the top correlated gene with *HOXD1* expression (left panel), whereas *HOXD1* was identified as the top correlated gene with *HOXD8* expression (right panel). Spearman's correlation coefficients, p-values, and q-values are indicated.

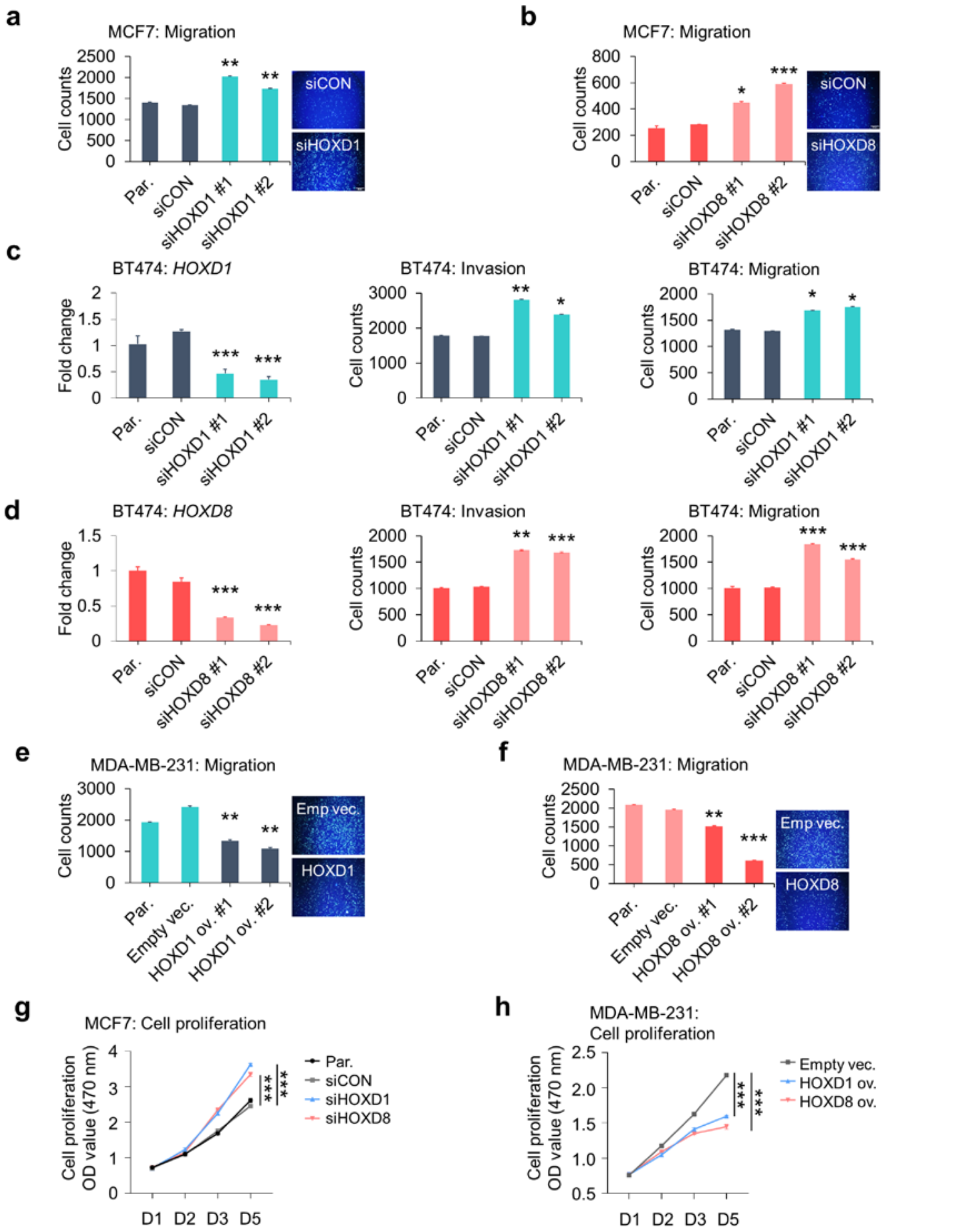

Supplementary Fig. 3. Functional effects of *HOXD1* and *HOXD8* perturbation on breast cancer cell invasion, migration, and proliferation. a, Transwell assays of MCF7 cells transfected with siCON or siHOXD1. **b,** Transwell assays of MCF7 cells transfected with

siCON or siHOXD8. **c,** Expression level of knocked-down *HOXD1* in BT474 breast cancer cells (left panel). The invaded (middle panel) and migrated (right panel) cells were stained with DAPI and counted. **d,** Expression level of knocked-down *HOXD8* in BT474 breast cancer cells (left panel). The invaded (middle panel) and migrated (right panel) cells were stained with DAPI and counted. **e,** Transwell assays of MDA-MB-231 cells transfected with empty vector or pHOXD1 plasmid DNA. **f,** Transwell assays of MDA-MB-231 cells transfected with empty vector or pHOXD8 plasmid DNA. **g**, Cell proliferation assay of MCF7 cells transfected with siCON, siHOXD1, or siHOXD8. **h**, Cell proliferation assay of MDA-MB-231 cells transfected with empty vector, pHOXD1, or pHOXD8 plasmid DNA.

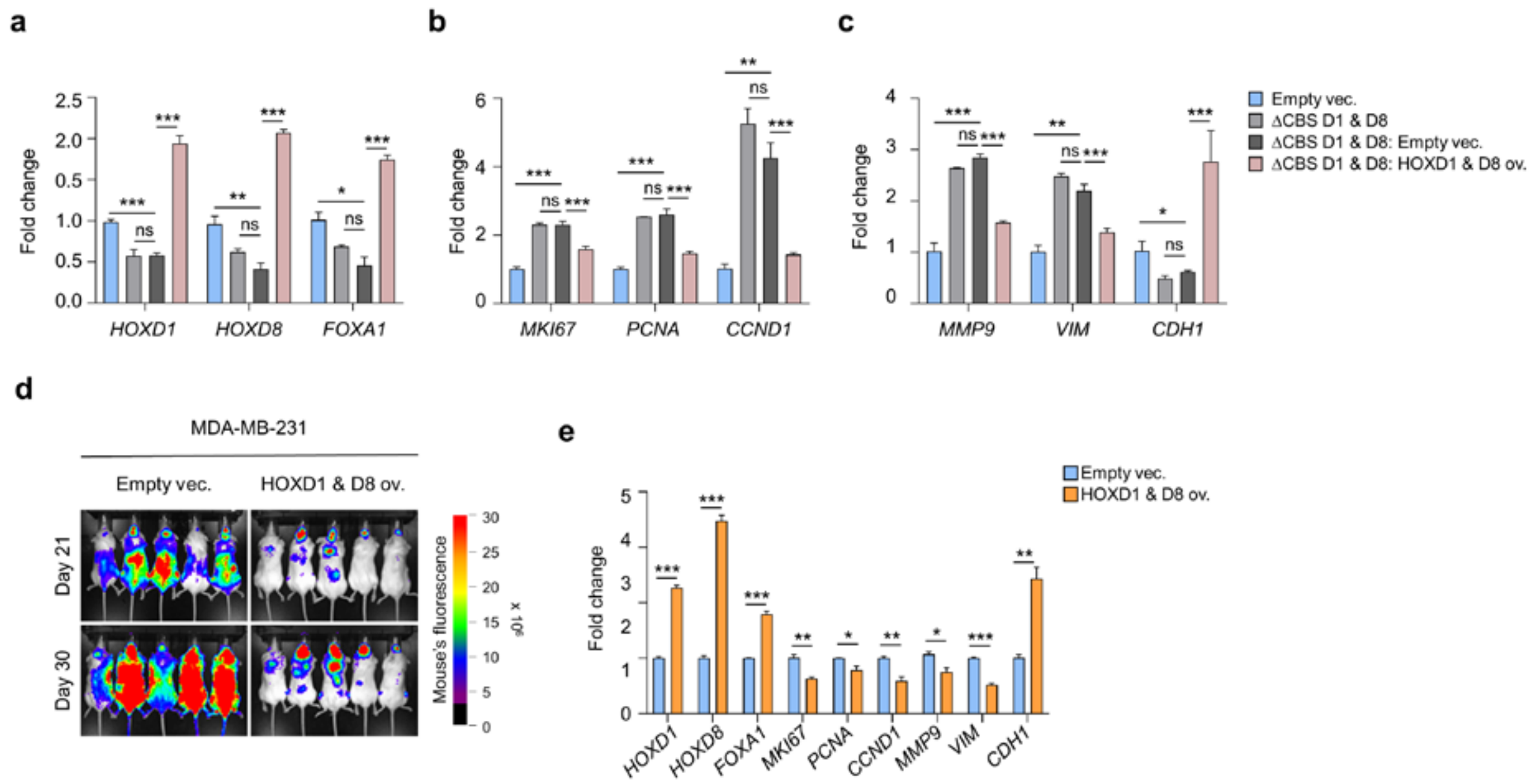

Supplementary Fig. 4. Molecular validation of HOXD1/HOXD8-associated tumor progression phenotypes in xenograft models. a-c, RT-qPCR analyses of *HOXD1*, *HOXD8*, and *FOXA1* expression (a), proliferation-associated genes (*MKI67*, *PCNA*, and *CCND1*) (b), and invasion/EMT-associated genes (*MMP9*, *VIM*, and *CDH1*) (c) in xenograft tumor tissues collected 40 days after implantation from Empty vec., ΔCBS D1 & D8, ΔCBS D1 & D8: Empty vec., and ΔCBS D1 & D8: HOXD1 & D8 ov. groups. **d,** Representative IVIS images of mice xenografted with MDA-MB-231 cells transfected with empty vector or HOXD1 & D8 overexpression vectors at days 21 and 30 after implantation. **e,** RT-qPCR analyses of *HOXD1*, *HOXD8*, *FOXA1*, *MKI67*, *PCNA*, *CCND1*, *MMP9*, *VIM*, and *CDH1* in xenograft tumor tissues collected 40 days after implantation from MDA-MB-231 empty vector and HOXD1 & D8 overexpression groups. Data are presented as mean ± SEM.

High expression in ... | Significant only

Gene	Cytoband	μ in Altered group	μ in Unaltered group	σ in Altered group	σ in Unaltered group	Log Ratio	p-Value	q-Value	Higher expression in
HOXD1	2q31.1	1.67	-0.39	0.88	0.86	2.06	1.98e-27	3.40e-23	Altered group
HOXD8	2q31.1	2.50	1.08	1.28	1.14	1.42	7.58e-12	6.52e-8	Altered group
CXCL9	4q21.1	6.47	5.79	0.92	1.31	0.68	1.538e-6	2.203e-3	Altered group
HOXD3	2q31.1	0.29	-0.41	1.00	0.79	0.70	1.795e-6	2.374e-3	Altered group
ERGIC1	5q35.1	0.81	0.43	0.55	0.61	0.38	3.798e-6	3.581e-3	Altered group
HOXC11	12q13.13	0.94	0.23	1.04	1.17	0.72	3.957e-6	3.581e-3	Altered group
HOXC10	12q13.13	1.49	0.72	1.17	1.45	0.77	1.138e-5	7.823e-3	Altered group
TFF3	21q22.3	2.46	1.60	1.25	2.09	0.85	1.442e-5	9.535e-3	Altered group
GZMK	5q11.2	4.25	3.79	0.72	1.06	0.46	2.744e-5	0.0136	Altered group
SDS	12q24.13	3.27	2.93	0.54	0.66	0.34	2.830e-5	0.0136	Altered group
HTRA4	8p11.22	2.27	1.63	1.08	1.14	0.64	3.553e-5	0.0161	Altered group
KLF4	9q31.2	-0.12	-0.61	0.82	0.87	0.49	4.080e-5	0.0171	Altered group
FOXA1	14q21.1	2.92	2.04	1.31	2.68	0.88	4.315e-5	0.0177	Altered group
MID1IP1	Xp11.4	-0.13	-0.40	0.46	0.54	0.27	6.225e-5	0.0234	Altered group
CEACAM7	19q13.2	1.84	1.06	1.37	1.10	0.79	6.260e-5	0.0234	Altered group
TIGIT	3q13.31	3.70	3.07	1.07	1.32	0.63	7.089e-5	0.0244	Altered group
CEACAM5	19q13.2	0.56	-0.68	2.30	1.92	1.23	1.680e-4	0.0444	Altered group
CEACAM6	19q13.2	3.01	1.44	2.91	3.12	1.57	1.959e-4	0.0470	Altered group
USP3	15q22.31	-0.02	-0.27	0.44	0.53	0.24	1.967e-4	0.0470	Altered group

Supplementary Fig. 5. Identification of genes positively correlated with coordinated HOXD1/HOXD8 expression in breast cancer. Differential gene expression analysis comparing *HOXD1*/*HOXD8*-altered and unaltered breast cancer tissues from the TCGA dataset (GSE65194). Genes showing coordinated expression changes associated with *HOXD1*/*HOXD8* alteration were identified using a two-fold change cut-off and statistical significance filtering.

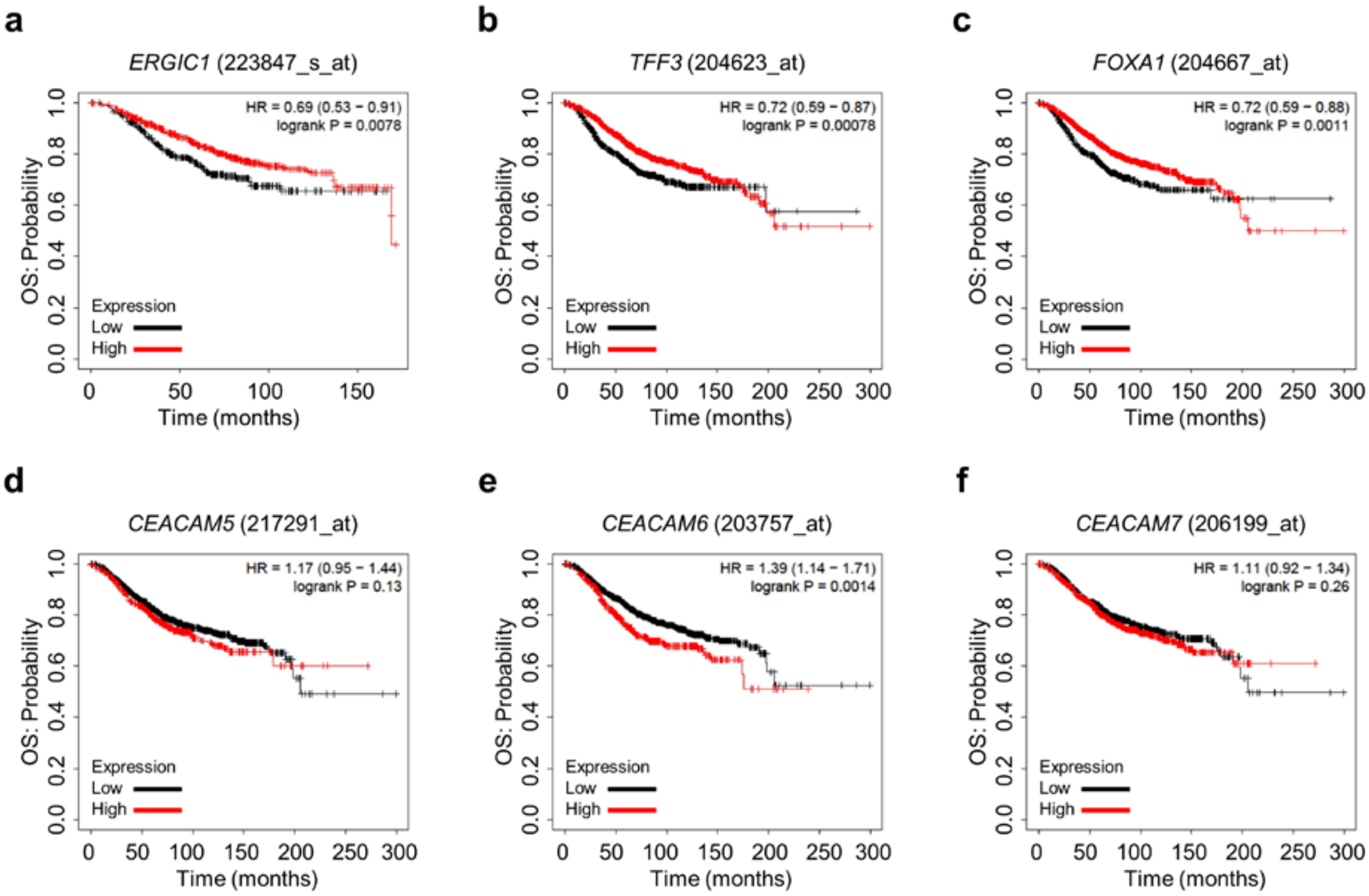

Supplementary Fig. 6. Kaplan–Meier survival analyses of HOXD1/HOXD8-associated downstream target genes in breast cancer patients. a-f, Kaplan–Meier overall survival (OS) analyses of breast cancer patients stratified by *ERGIC1*, *TFF3*, *FOXA1*, *CEACAM5*, *CEACAM6*, and *CEACAM7* expression levels, respectively. Patients were divided into low- and high-expression groups using the upper quartile cutoff. Log-rank P-values and hazard ratios (HR) are indicated in each panel.
